# Antenatal Screening for Down Syndrome Using Serum Placental Growth Factor with the Combined, Quadruple, Serum Integrated and Integrated Tests

**DOI:** 10.1371/journal.pone.0046955

**Published:** 2012-10-03

**Authors:** Nicholas J. Wald, Jonathan P. Bestwick, Lynne M. George, Wayne J. Huttly

**Affiliations:** Wolfson Institute of Preventive Medicine, Barts and the London School of Medicine and Dentistry, Queen Mary University of London, London, United Kingdom; The University of Texas M. D. Anderson Cancer Center, United States of America

## Abstract

**Objective:**

To estimate the value of first or second trimester placental growth factor (PlGF) as an additional antenatal screening marker for Down syndrome.

**Design:**

Nested case-control study.

**Setting:**

Antenatal screening service.

**Population or Sample:**

532 Down syndrome pregnancies and 1,155 matched unaffected pregnancies.

**Methods:**

Stored maternal serum samples (−40°C) were assayed for PlGF. Monte Carlo simulation was used to estimate the screening performance of PlGF with the Combined, Quadruple, serum Integrated and Integrated tests.

**Main Outcome Measures:**

Median PlGF levels in affected and unaffected pregnancies and screening performance (detection rates [DR] for specified false-positive rates [FPR] and *vice versa*).

**Results:**

First trimester median PlGF was 15%, 28% and 39% lower in Down syndrome than unaffected pregnancies at 11, 12 and 13 completed weeks’ gestation respectively (all p<0.001). Second trimester median PlGF was 31% lower at 14 weeks (p<0.001), and the difference decreased (6% lower at 17 weeks). At a 90% DR with first trimester markers measured at 13 weeks, adding PlGF decreased the FPR from 11.1 to 5.1% using the Combined test, 9.3% to 4.5% using the serum Integrated test, and 3.4% to 1.5% using the Integrated test (or 1.5 to 1.4% with first trimester markers measured at 11 weeks). Adding PlGF to the Quadruple test (measured at 15 weeks) decreased the FPR from 10.0% to 9.6% at a 90% DR.

**Conclusions:**

First trimester PlGF measurements improve the performance of antenatal screening for Down syndrome using the Combined, serum Integrated and Integrated tests. Second trimester PlGF measurements are of limited value.

## Introduction

Placental growth factor (PlGF) is an angiogenic hormone which has been shown to be useful in late first trimester and early second trimester antenatal screening for pre-eclampsia. First trimester levels were found to be 36% lower in women who developed early pre-eclampsia compared to unaffected controls [1] and early second trimester levels were 30% lower. [2] The value of PlGF in screening for Down syndrome is less clear. Five studies have reported results on PlGF measured in the late first trimester (11 to 13 weeks’ gestation). Four of these showed, on average, reduced PlGF levels in Down syndrome compared with unaffected pregnancies (22% [3], 24% [4], 29% [5] and 38% [6] lower based on 91, 70, 90 and 42 affected pregnancies respectively) and one study found a higher level in affected pregnancies (26% higher based on 45 affected pregnancies [7]). In the *second trimester* three studies reported PlGF results; one showed reduced levels (33% lower based on 24 affected prengnancies [8]), one increased levels (42% higher based on 36 affected pregnancies [9]) and one no material difference in Down syndrome compared with unaffected pregnancies (1% higher based on 39 affected pregnancies [10]).

The uncertain value of PlGF in antenatal screening for Down syndrome prompted us to investigate the matter further by comparing the screening performance with and without (i) first trimester PlGF measurements added to the Combined test (nuchal translucency [NT], free β-human chorionic gonadotrophin [hCG] and pregnancy associated plasma protein A [PAPP-A] measured between 11 and 13 weeks’ gestation), (ii) second trimester PlGF measurements added to the early second trimester Quadruple test (alphafetoprotein [AFP], unconjugated oestriol [uE_3_], free β-hCG and inhibin-A measured between 14 and 22 weeks’ gestation), (iii) first trimester PlGF measurements added to the Integrated test (first trimester NT and PAPP-A and second trimester AFP, uE3 free β-hCG and Inhibin-A) and serum Integrated test (Integrated test without NT) and (iv) early second trimester PlGF measurements added to the Integrated and serum Integrated tests.

## Methods

According to guidelines from the National Research Ethics Committee, our research does not require research ethics committee approval as serum samples were collected as part of a routine antenatal screening programme. Women are informed of the possible subsequent use of samples in research or screening programme audits, so they could indicate to the programme staff that they did not want their samples used. The research analysis was conducted on anonymised samples and data.

We identified 532 Down syndrome singleton pregnancies screened at the Wolfson Insititute of Preventive Medicine between February 2000 and May 2010 from the screening service records and by linkage to data from the National Cytogenetic Register held at the Wolfson Institute. Among the 532 affected pregnancies, 289 were screened using the Combined test (from February 2005), 217 using the Quadruple test and 26 using the Integrated test (from March 2003). Each affected pregnancy was matched with 2 (Combined and Quadruple test) or 5 (Integrated test) unaffected control pregnancies for gestational age (same day), maternal age (in 5 year categories) and length of storage (in six-month categories).

Frozen (−40°C) stored samples were thawed and assayed for placental growth factor using the AutoDELPHIA® PlGF kit (Perkin Elmer). The samples were assayed “blind” i.e. without knowledge of whether the samples were from a Down syndrome or unaffected pregnancy. The inter-assay coefficient of variation was 7.5% and intra-assay coefficient of variation 3.9%. Serum from women screened using the Combined and Integrated tests was used to assess first trimester PlGF, and serum from women screened using the Quadruple and Integrated tests was used to assess second trimester PlGF.

PlGF concentrations were expressed as multiples of the median (MoM) for unaffected pregnancies of the same gestational age by performing a regression of the log median PlGF against the median gestational age in 2-day categories for first trimester measurements and weekly categories for second trimester measurements (weighted by the number of women in each category) and dividing PlGF concentrations by the regressed (i.e. expected) concentration for the same gestational age. MoM values were adjusted for maternal weight by performing a regression of the log median MoM values against weight in unaffected pregnancies and dividing MoM values by the regressed value for the same weight. Associations between weight adjusted PlGF MoM values and maternal smoking and ethnicity were also determined and MoM values further adjusted as required. The change in median MoM in Down syndrome pregnancies was investigated by performing a regression of the median PlGF MoM against the median gestational age in 2-day categories for first trimester measurements and weekly categories for second trimester measurements (weighted by the number of women in each category; 19 to 22 weeks were combined into one category due to the small numbers of Down syndrome pregnancies with PlGF measurements beyond 18 weeks’ gestation). Probability plots and, if appropriate, the approximate point of risk reversal (to ensure risk estimation is a monotonic function of the marker value [11]) were used to specify truncation limits within which the range of values for PlGF approximately followed log-Gaussian distributions in affected and unaffected pregnancies.

Median PlGF MoM values were used as the measure of central tendency to avoid the influence of outliers and their log values as estimates of the means. Standard deviations (log) were calculated by regression of the points on the probability plot between the 10^th^ and 90^th^ centiles and correlation coefficients with the standard Down syndrome screening markers (first trimester NT, free β-hCG and PAPP-A and second trimester AFP, uE3 free β-hCG and Inhibin-A) were calculated after excluding points more than 3.5 standard deviations from the mean (correlations between first trimester PlGF and second trimester markers, and second trimester PlGF and first trimester markers were estimated using data from women screened using the Integrated test). Log means and standard deviations of the standard Down syndrome screening markers and correlation coefficients were taken from the SURUSS report.[12]–[15].

Monte Carlo simulation was used to estimate screening performance instead of numerical integration as used previously[12]–[15] (because Monte Carlo simulation is computationally faster). Hypothetical random samples of 250,000 Down syndrome and 250,000 unaffected pregnancies were generated based on the specified Gaussian distributions. Each simulated pregnancy was assigned a maternal age based on the maternal age distribution of maternities in England and Wales 2006–2008 [16] (instead of 1996–1998 as used previously, so screening performance figures of tests without PlGF will be expected to be a little different from those previously reported.[12]–[15]) and the maternal age-specific odds of an affected livebirth. [17], [18] For each simulated pregnancy, the predicted risk of having a pregnancy with Down syndrome in the early second trimester was calculated by multiplying the maternal age specific odds of having an affected live birth adjusted to early mid-trimester by multiplying by 1/0.77 to allow for the general fetal loss in Down syndrome pregnancies from this time in pregnancy until term [19] by the likelihood ratio (for a given set of marker values) obtained from the overlapping multivariate Gaussian distributions of marker levels in affected and unaffected pregnancies. A woman was classified as screen positive if her risk of having a pregnancy affected with Down syndrome was greater than or equal to a specified risk cut-off level. Screening performance estimates were calculated as the detection rate (DR) for false-positive rates (FPR) of 1, 3 and 5%, the FPR for DR’s of 85, 90 and 95% and the DR and FPR for early second trimester risk cut-offs of 1 in 100, 1 in 150 and 1 in 200.

## Results

Table 1 shows the number of pregnancies screened classified according to test, gestational age when screened, and selected characteristics of the Down syndrome and unaffected pregnancies. The distributions of the variables in the two groups were similar.

**Table 1:** Number of pregnancies classified according to screening test, gestational age when screened, and selected characteristics of the Down syndrome and unaffected pregnancies.

	Down syndrome (N = 532)	Unaffected (N = 1,152)
Screening test received		
Combined test	289	576
Quadruple test	217	433
Integrated test	26	143
Median gestational age (days)		
First trimester	88	88
Second trimester	114	114
Median maternal age at EDD	37	37
Median maternal weight (kg)	66	66
Smoking (%)	5.8	7.7
Ethnicity (%)		
Afro-Caribbean	9.2	10
White	72	73
South Asian	6.5	5.7
Oriental	2.6	3.3
Other	10.2	8.3

EDD: expected date of delivery.

**Figure 1:**
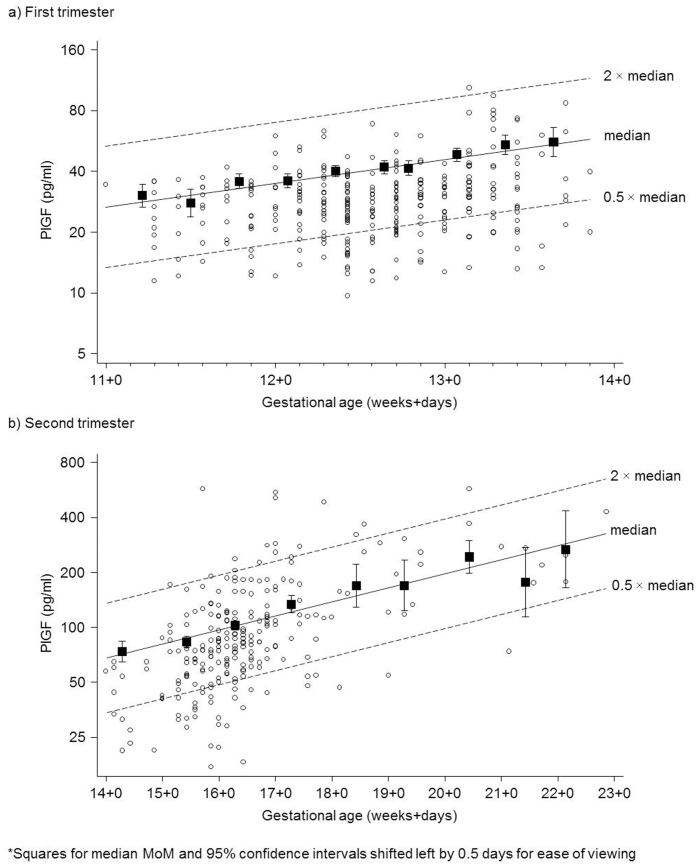
First trimester (a) and second trimester (b) placental growth factor (PlGF) according to gestational age in Down syndrome pregnancies (circles) and medians in 2-day intervals* (a) and weekly intervals (b) in unaffected pregnancies (squares, with 95% confidence intervals) together with expected (regressed) median in unaffected pregnancies.

Figure 1a shows the concentration of late first trimester (11–13 completed weeks) PlGF in affected pregnancies according to gestational age together with the expected (regressed) median concentration in unaffected pregnancies. The median PlGF in unaffected pregnancies increased by 31% per week of gestation (p<0.001). Figure 1b shows the corresponding data for early second trimester PlGF (14–22 completed weeks). The median PlGF in unaffected pregnancies increased by 19% per week of gestation (p<0.001). In unaffected pregnancies, first trimester PlGF MoM values decreased by 1.3% per 5 kg increase in maternal weight (p = 0.031) and second trimester PlGF MoM values decreased by 3.1% per 5 kg increase (p = 0.002);

Smokers had higher weight adjusted PlGF MoM values than non-smokers; 32% higher in the first trimester (95% confidence interval 25% to 61%) and 36% higher in the second trimester (95% confidence interval 11% to 59%). First trimester PlGF MoM values were 18% higher in Afro-Caribbean women compared with white women (95% confidence interval 7% to 35%) and second trimester MoM values 30% higher (95% confidence interval 9% to 54%). First trimester weight adjusted PlGF MoM values were therefore divided by 1.32 in smokers and by 1.18 in Afro-Caribbean women; second trimester MoM values by 1.36 in smokers and 1.30 in Afro-Caribbean women. There were no significant increases or decreases in either first or second trimester weight adjusted PlGF MoM values in other ethnic groups.

**Figure 2:**
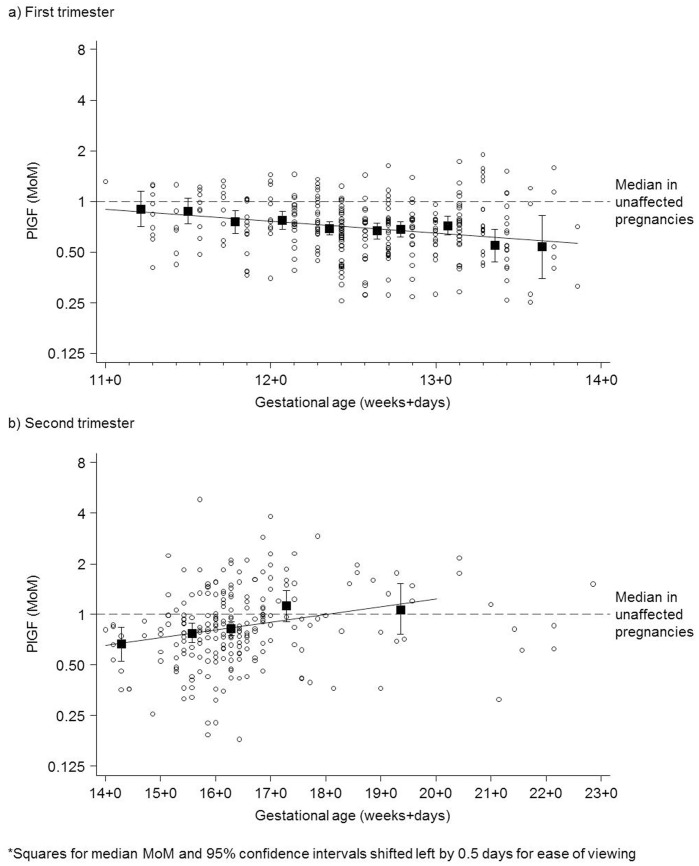
First trimester (a) and second trimester (b) placental growth factor (PlGF) maternal weight, smoking and ethnicity adjusted MoM values according to gestational age and medians (squares, with 95% confidence intervals) in 2-day intervals* (a) and weekly intervals (b) in Down syndrome pregnancies together with expected (regressed) median.

Figure 2a shows the first trimester PlGF MoM values (after adjustment for maternal weight, smoking and Afro-Caribbean ethnicity) in Down syndrome pregnancies according to gestational age together with the expected (regressed) median MoM and the corresponding results for second trimester PlGF. The median MoM values decreased by 15% per week (p = 0.003), with the median MoM at 11, 12 and 13 completed weeks’ gestation being 0.85 (95% confidence interval, CI 0.76 to 0.95), 0.72 (95% CI 0.68 to 0.76) and 0.61 (95% CI 0.55 to 0.67) respectively. Table 2 shows the observed and regressed median MoM values at each completed week of gestation.

**Table 2:** Observed and regressed median PlGF MoM in Down syndrome pregnancies according to gestational age.

Completed week of gestation	Number of affected pregnancies	Observed	Regressed	
		Median MoM	Median MoM (95% CI)*	p-value
First trimester				
11	48	0.82	0.85 (0.76 to 0.95)	0.011
12	164	0.71	0.72 (0.68 to 0.76)	<0.001
13	89	0.68	0.61 (0.55 to 0.67)	<0.001
Second trimester				
14	13	0.66	0.69 (0.54 to 0.89)	0.019
15	63	0.77	0.77 (0.65 to 0.91)	0.016
16	97	0.82	0.85 (0.75 to 0.97)	0.031
17	27	1.12	0.94 (0.80 to 1.12)	0.368
18–22	24	1.06	1.15 (0.82 to 1.62)**	0.279

* Median MoM at completed week (week+3 days).

** Regressed median MoM at 135.5 days gestation (median gestational at 18–22 completed weeks).

Figure 2b and table 2 show that the median second trimester MoM increased by 11% per week (p<0.001), with the median MoM at 14, 15, 16 completed weeks’ gestation being 0.69 (95% CI 0.54 to 0.89), 0.77 (95% CI 0.65 to 0.91) and 0.85 (95% CI 0.75 to 0.97) respectively. The median MoM at 17, completed weeks’ gestation was 0.94 (95% CI 0.80 to 1.12) and at 18–22 weeks was 1.15 (95% CI 0.82 to 1.62); neither statistically significantly different from the median in unaffected pregnancies (1.0 MoM). Therefore, screening performance estimates for PlGF were not considered for measurements of PlGF beyond 16 weeks’ gestation.

Inspection of probability plots of PlGF MoM values in Down syndrome and unaffected pregnancies showed that the distributions were reasonably Gaussian between 0.4 to 2.5 MoM (see figure in Appendix S1). The standard deviations of the first and second trimester log PlGF MoM values were, respectively, 0.1705 and 0.2243 in affected pregnancies and 0.1556 and 0.1786 in unaffected pregnancies. As a single marker the PlGF detection rates for a 5% false-positive rate were 14%, 26% and 41% at 11, 12 and 13 completed weeks respectively and 27%, 21% and 16% at 14, 15 and 16 weeks.

Table 3 shows the screening performance of the Combined, Quadruple, serum Integrated and Integrated tests with and without the addition of PlGF at specified detection rates and at specified false-positive rates. The addition of a *first trimester* measurement of PlGF improves the screening performance of the Combined test; at a 90% detection rate the false-positive rate decreased from 6.7% to 6.1% with markers measured at 11 completed weeks of gestation and from 11.1% to 5.1% at 13 completed weeks’ gestation. The addition of a *second trimester* PlGF measurement to the Quadruple test had little influence on screening performance; for example, at a 90% detection rate the false-positive rate decreased from 10.0% to 9.6% if PlGF is measured at 15 completed weeks’ gestation. Results with PlGF measured at 14 and 16 completed weeks’ gestation are similar [9.4% and 9.7% respectively] and so second trimester screening performance estimates are not shown in the Table.

**Table 3:** Screening performance according to detection and false-positive rates of the Combined, Quadruple and Integrated tests with and without the addition of first or second trimester placental growth factor (PlGF) according to gestational age markers are measured.

	Gestational age first trimester markers measured (completed weeks)	DR (%) for FPR of:-			FPR (%) for DR of:-		
Test		1%	3%	5%	85%	90%	95%
Comined							
Without PlGF	11	76	84	88	3.2	6.7	16.6
	12	74	83	86	4.2	8.6	19.8
	13	70	79	84	5.9	11.1	23.0
With PlGF	11	77	85	89	2.9	6.1	15.3
	12	77	85	89	2.8	6.1	14.9
	13	78	86	90	2.4	5.1	12.4
Quadruple							
Without PlGF	–	64	77	83	5.9	10.0	19.6
With PlGF at 15	–	66	78	84	5.7	9.6	18.9
completed weeks							
Serum Integrated							
Without PlGF	11	72	83	87	3.8	7.2	15.4
	12	69	81	86	4.7	8.4	17.3
	13	67	79	84	5.3	9.3	18.6
With first	11	73	84	88	3.4	6.5	13.9
trimester PlGF	12	73	84	88	3.4	6.1	12.9
	13	76	87	91	2.5	4.5	9.7
With second	11	74	84	88	3.3	6.4	14.1
trimester PlGF at 15	12	71	82	87	4.1	7.6	16.0
completed weeks	13	69	81	86	4.7	8.5	17.2
Integrated							
Without PlGF	11	88	93	95	0.6	1.5	5.0
	12	86	92	94	0.8	2.2	6.6
	13	82	89	92	1.5	3.4	9.1
With first	11	88	93	95	0.5	1.4	4.5
trimester PlGF	12	88	93	95	0.6	1.5	4.6
	13	88	94	96	0.6	1.5	4.2
With second	11	89	94	95	0.5	1.3	4.4
trimester PlGF at 15	12	87	92	94	0.7	1.8	5.8
completed weeks	13	84	90	93	1.3	2.9	8.1

Table 3 shows that at a 90% detection rate the addition of *first trimester* PlGF measurement to the Integrated test reduces the false-positive rate from 1.5% to 1.4% at 11 completed weeks’ gestation and from 3.4% to 1.5% at 13 completed weeks. The addition of a *second trimester* PlGF measurement at 15 completed weeks’ gestation to the Integrated test reduces the false positive rate from 1.5% to 1.3% when first trimester markers are measured at 11 completed weeks’ gestation and from 3.4% to 2.9% when first trimester markers are measured at 13 completed weeks. The results are similar if PlGF is measured at 14 or 16 completed weeks’ gestation. As with the Integrated test, the addition of a first trimester PlGF measurement to the serum Integrated test improves screening performance but a second trimester PlGF has little effect (Table 3). Table 4 shows, in a similar way to Table 3, results according to risk cut-off. Table 5 shows the overall screening performance (first trimester markers measured at 11–13 weeks’ gestation) with and without the addition of first and second trimester PlGF to the Combined, Serum Integrated and Integrated tests assuming equal numbers of women screened at each week of gestation in the first trimester. The addition of a first trimester PlGF measurement decreases the overall false-positive rate by about one third. For example, at a 90% detection rate, the overall Combined test false-positive rate decreases from 8.8% to 5.8% and the overall Integrated test false-positive rate from 8.3% to 5.7%.

**Table 4:** Screening performance according to risk cut-off of the Combined, Quadruple and Integrated tests with and without the addition of first or second trimester placental growth factor (PlGF) according to gestational age markers are measured.

	Gestational age first trimester markers measured (completed weeks)	Risk cut-off (early second trimester)								
		1 in 100			1 in 150			1 in 200		
Test		DR (%)	FPR (%)	OAPR	DR (%)	FPR (%)	OAPR	DR (%)	FPR (%)	OAPR
Comined										
Without PlGF	11	82	2.0	1∶9	84	3.0	1∶12	86	3.9	1∶16
	12	80	2.1	1∶9	83	3.0	1∶13	85	3.9	1∶16
	13	77	2.3	1∶11	81	3.5	1∶15	83	4.6	1∶19
With PlGF	11	82	1.9	1∶8	85	2.8	1∶12	87	3.7	1∶15
	12	82	1.9	1∶8	85	2.7	1∶11	87	3.6	1∶15
	13	83	1.9	1∶8	86	2.8	1∶11	88	3.5	1∶14
Quadruple										
Without PlGF	–	78	3.2	1∶14	82	4.6	1∶19	85	5.8	1∶24
With PlGF at 15	–	78	3.0	1∶13	82	4.2	1∶18	85	5.5	1∶23
completed weeks										
Serum Integrated										
Without PlGF	11	81	2.5	1∶11	85	3.6	1∶15	87	4.6	1∶19
	12	80	2.7	1∶12	83	3.9	1∶16	86	5.0	1∶20
	13	79	2.9	1∶13	83	4.2	1∶18	85	5.4	1∶22
With first	11	82	2.5	1∶11	85	3.6	1∶15	87	4.6	1∶18
trimester PlGF	12	83	2.6	1∶11	86	3.7	1∶15	88	4.7	1∶19
	13	85	2.4	1∶10	88	3.4	1∶14	90	4.3	1∶17
With second	11	82	2.3	1∶10	85	3.3	1∶14	87	4.3	1∶17
trimester PlGF at 15	12	81	2.5	1∶11	84	3.6	1∶15	86	4.7	1∶19
completed weeks	13	80	2.6	1∶12	83	3.9	1∶16	85	4.9	1∶20
Integrated										
Without PlGF	11	89	1.3	1∶5	91	1.9	1∶7	92	2.4	1∶9
	12	88	1.4	1∶6	90	2.0	1∶8	91	2.6	1∶10
	13	86	1.6	1∶7	88	2.4	1∶10	89	3.0	1∶12
With first	11	90	1.3	1∶5	91	1.8	1∶7	92	2.3	1∶9
trimester PlGF	12	89	1.3	1∶5	91	1.9	1∶7	92	2.4	1∶9
	13	90	1.4	1∶5	91	1.9	1∶7	93	2.4	1∶9
With second	11	89	1.2	1∶5	91	1.7	1∶7	92	2.2	1∶8
trimester PlGF at 15	12	88	1.3	1∶5	90	1.9	1∶7	91	2.4	1∶9
completed weeks	13	86	1.5	1∶6	88	2.2	1∶9	90	2.8	1∶11

**Table 5:** Screening performance according to detection and false-positive rates of the Combined, Quadruple and Integrated tests with and without the addition of first trimester (11–13 weeks^1^) or second trimester placental growth factor (PlGF).

	DR (%) for FPR of:-			FPR (%) for DR of:-		
Test	1%	3%	5%	85%	90%	95%
Combined						
Without PlGF	73	82	86	4.4	8.8	19.8
With PlGF	77	85	89	2.7	5.8	14.2
Serum Integrated						
Without PlGF	69	81	86	4.6	8.3	17.1
With first trimester PlGF	74	85	89	3.1	5.7	12.2
With second trimester PlGF	71	82	87	4.0	7.5	15.8
Integrated						
Without PlGF	85	91	94	1.0	2.4	6.9
With first trimester PlGF	88	93	95	0.6	1.5	4.4
With second trimester PlGF	87	92	94	0.8	2.0	6.1

1 Average of the individual estimates with first trimester markers measured at 11, 12 and 13 completed weeks of gestation (see Table 3), assuming equal numbers of women screened at each week.

Probability plots of PlGF MoM values and the statistical parameters (means standard deviations, correlation coefficients and truncation limits) used in this study for the estimation of screening performance are shown in the Appendix S1.

## Discussion

In the late first trimester our results show that PlGF levels are reduced in Down syndrome pregnancies at 11, 12 and 13 completed weeks of gestation. The levels are lower at 12 than 11 weeks and lower at 13 than at 12 weeks. Correspondingly, the improvement in screening performance due to the addition of PlGF to the Combined, serum Integrated and Integrated tests increases over these weeks. For example, with the Combined test, the addition of PlGF at 11 completed weeks’ of pregnancy decreases the false-positive rate at a 90% detection rate by 0.6 percentage points (6.1% v 6.7%) but by 6 percentage points at 13 weeks (5.1% v 11.1%). Other first trimester studies on PlGF do not report results by individual weeks. Their overall results are consistent with ours in four studies[3]–[6], but not in one. [7].

The screening performance of the Combined (and also the serum Integrated test) is best if the first trimester markers, including PlGF, are measured at 13 completed weeks. However, the performance of the Integrated test is similar when the first trimester markers, including PlGF, are measured at 11, 12 or 13 completed weeks; the addition of PlGF thus removes the advantage of performing the first stage of an Integrated test at 11 weeks compared with 12 or 13 weeks.

In the early second trimester of pregnancy PlGF values in Down syndrome pregnancies tend to be reduced, but increase towards the normal median with increasing gestation, possibly increasing above the median and the standard deviation of second trimester values is greater than that of first trimester values. Both of these effects mean that the measurement of PlGF in the second trimester does not offer a clinically significant improvement in screening performance.

We used a linear regression model in the first trimester and a separate linear regression in the second trimester to quantify the PlGF levels in affected pregnancies according to gestational age; both regression models fitted the data reasonably well (see Figure 2), and fitted better than a single quadratic regression using all the data together. Separate linear regressions also have the advantage of being more stable.

PlGF has been shown to be a useful marker in prenatal screening for pre-eclampsia in both the first and second trimesters of pregnancy. [1], [2] A first trimester PlGF measurement could be used as part of the Combined, serum Integrated or Integrated tests for Down syndrome as well as in screening for pre-eclampsia. There is some benefit for a second trimester measurement of PlGF if women book too late for a Combined, serum Integrated or Integrated test even though the improvement in the performance of antenatal screening for Down syndrome is small.

In estimating screening performance using first trimester markers alone compared with that using the Integrated test markers, there can be bias if the first trimester markers are associated with miscarriage at about 10–14 weeks. This, for example, affects PAPP-A, in which low values are associated with miscarriage as well as with Down syndrome. [12] Any effect of PlGF being associated with miscarriage in this period is likely to be small, if present at all, because in our data there wase no significant difference in the median PlGF MoM in affected pregnancies tested using the Combined test and those tested using the Integrated test (0.70 and 0.66 respectively, p = 0.35).

The financial cost of including first trimester PlGF measurement in the Combined, Serum Integrated and Integrated tests depends on when the first trimester markers are measured. If measured at 11 weeks, the cost per Down syndrome pregnancy diagnosed is about £300 more expensive with the Combined test and £600 more expensive with the Integrated test. Measured at 12 weeks, costs are £2,500 less for the Combined test and £100 less for the Integrated test; if measured at 13 weeks, the amounts are £7,100 and £1,700 less respectively. These estimates are based on using the unit costs given in SURUSS^12^ increased by 25% to allow for inflation. Overall, therefore, the addition of PlGF at 11–13 weeks is probably worthwhile, given the improvement in screening performance with little or no increased cost per Down syndrome pregnancy diagnosed.

### Conclusion

This study, based on 532 Down syndrome pregnancies, is the largest to have investigated the value of PlGF in antenatal screening for Down syndrome. It shows that PlGF is a useful Down syndrome screening marker in the late first trimester of pregnancy but of little value when measured in the early second trimester. Women having a Combined, serum Integrated or Integrated test should have PlGF measured in the first trimester and women having the Quadruple test need only have PlGF measured in the second trimester if the intention is to screen for pre-eclampsia as well as for Down syndrome.

## Supporting Information

Appendix S1 **PlGF probability plots and statistical parameters.**
(DOCX)
