# Supplementary material for: Antenatal Screening for Down Syndrome Using Serum Placental Growth Factor with the Combined, Quadruple, Serum Integrated and Integrated Tests
Source: PLoS One. 2012 Oct 3;7(10):e46955. doi: 10.1371/journal.pone.0046955 (PMC3463523; doi:10.1371/journal.pone.0046955)
Supplement: Appendix S1 — PlGF probability plots and statistical parameters. (DOCX) [file pone.0046955.s001.docx]

Appendix

Appendix figure 1 shows probability plots of placental growth factor in Down syndrome and unaffected pregnancies. Appendix Table 1 provides the statistical parameters (means, standard deviations, correlation coefficients and truncation limits) of PlGF in Down syndrome and unaffected –pregnancies.

Appendix figure 1: Probability plot for (a) first trimester and (b) second trimester PlGF in Down syndrome (squares) and unaffected (circles) pregnancies.

a) First trimester

Unaffected

Down syndrome

b) Second trimester

Unaffected

Down syndrome

**Appendix Table 1: Screening marker parameters (means, standard deviations, correlation coefficients and truncation limits of log MoM values) for placental growth factor (PlGF) in Down syndrome and unaffected pregnancies**

|  | Down syndrome | |  | Unaffected | |
| --- | --- | --- | --- | --- | --- |
| Median |  |  |  |  |  |
| First trimester^1^ |  |  |  |  |  |
| 11 completed weeks | -0.0714 | |  | 0 | |
| 12 completed weeks | -0.1439 | |  | 0 | |
| 13 completed weeks | -0.2164 | |  | 0 | |
| Second trimester^2^ |  | |  |  |  |
| 14 completed weeks | -0.1591 | |  | 0 | |
| 15 completed weeks | -0.1143 | |  | 0 | |
| 16 completed weeks | -0.0695 | |  | 0 | |
|  |  |  |  |  |  |
| Standard deviation |  |  |  |  |  |
| First trimester | 0.1705 | |  | 0.1556 | |
| Second trimester | 0.2243 | |  | 0.1786 | |
|  |  |  |  |  |  |
| Truncation limits (MoM) |  |  |  |  |  |
| First trimester | 0.40 to 2.50 | | | | |
| Second trimester^3^ |  |  |  |  |  |
| 14 completed weeks | 0. 40 to 1.89 | | | | |
| 15 completed weeks | 0. 40 to 1.58 | | | | |
| 16 completed weeks | 0. 40 to 1.32 | | | | |
|  |  |  |  |  |  |
| Correlation (with) | First trimester | Second trimester |  | First trimester | Second trimester |
| First trimester |  |  |  |  |  |
| NT | 0.0134 | 0.0327 |  | -0.0093 | -0.0623 |
| Free β-hCG | -0.0651 | - |  | 0.1526 | - |
| PAPP-A | 0.0424 | 0.0599 |  | 0.2860 | 0.3223 |
| PlGF | 1 | 0.4712 |  | 1 | 0.4643 |
|  |  |  |  |  |  |
| Second trimester |  |  |  |  |  |
| AFP | -0.2692 | 0.1973 |  | -0.1477 | 0.0204 |
| uE3 | -0.0056 | 0.2077 |  | -0.0547 | 0.1509 |
| Free β-hCG | -0.3058 | -0.2110 |  | -0.0854 | 0.0724 |
| Inhibin-A | -0.0053 | -0.2581 |  | -0.1509 | -0.0359 |
| PlGF | 0.4712 | 1 |  | 0.4643 | 1 |

^1^Median MoM in Down syndrome pregnancies = 10^0.7573536-0.0103592×gestational age in days^

^2^Median MoM in Down syndrome pregnancies = 10^-0.805727+0.0064023×gestational age in days^

^3^Upper truncation limit set at the point of risk reversal
